# Methylation Pattern of the SOCS3 and IL6R Promoters in Rheumatoid Arthritis

**DOI:** 10.1155/2020/8394659

**Published:** 2020-03-31

**Authors:** Marek Cieśla, Bogdan Kolarz, Maria Majdan, Dorota Darmochwał-Kolarz

**Affiliations:** ^1^Institute of Medical Sciences, Medical College, University of Rzeszow, 35-959 Rzeszow, Poland; ^2^Department of Rheumatology and Connective Tissue Diseases, Medical University of Lublin, 20-090 Lublin, Poland

## Abstract

Interleukin-6 (IL-6) plays an essential function in the development of rheumatoid arthritis (RA), mainly through its proinflammatory effect, which may lead to joint destruction. The genes encoding IL-6 receptor (*IL6R*) and suppressor of cytokine signaling 3 (*SOCS3*) play a key role in the IL-6 signaling pathway, but their epigenetic regulation remains unclear. The aim of the study was to investigate how the presence of methylation in the *SOCS3* and *IL6R* promoters is associated with the morbidity and severity of RA. A total of 146 unrelated individuals, 122 with RA and 24 healthy controls, were enrolled in the study. All subjects were genotyped with regard to the rs4969168 and rs4969170 polymorphisms in the *SOCS3* gene and the rs2228145 and rs4129267 polymorphisms in *IL6R*. The methylation study included 52 patients with RA and 24 healthy controls. Qualitative real-time methylation-specific PCR was used to evaluate methylation status. We found no differences between patients and healthy controls in the methylation pattern in the *IL6R* and *SOCS3* promoter regions and in variants frequency. The methylation profiles of the *SOCS3* and *IL6R* promoters do not support the hypothesis that the genes *SOCS3* and *IL6R* involved in the JAK-STAT signaling pathway are epigenetically deregulated in whole blood.

## 1. Introduction

Rheumatoid arthritis (RA) is a chronic inflammatory disease that leads to joint destruction [1, 2]. Single nucleotide polymorphisms (SNPs) are considered important genetic risk factors in the development of RA [3, 4]. Apart from SNPs, epigenetic modifications such as DNA methylation have been reported as an important factor related to the development of a number of diseases [5]. The presence of DNA methylation manifested by the presence of 5-methylcytosine in the CpG dinucleotide is considered an important mechanism regulating gene expression. In most cases, the presence of a large percentage of CpGs in the promoter region is associated with constant gene silencing, but recent reports have demonstrated that the methylation pattern in regulatory regions such as transcription factor binding sites or gene enhancers may play a critical role in this regulation [5, 6]. Despite these findings, molecular mechanisms involved in RA pathogenesis are still unclear [3].

Interleukin-6 (IL-6) is a cytokine responsible for both inflammation and hematopoiesis. IL-6 stimulates the production of hepatic acute-phase proteins such as C-reactive protein (CRP) and plays a critical function in joint destruction caused by inflammation, thereby affecting RA development [7]. The IL-6 signaling pathway is mediated by the membrane-bound IL-6 receptor (IL6R), which consists of two glycoproteins: IL6R*α* and *β* (gp130). The latter is expressed by most cells, while expression of IL6R*α* on the cell surface is limited [8]. However, proteolysis of IL6R or alternative mRNA splicing may generate a soluble form of IL6R (sIL6R). In a transsignaling process, activated sIL6R binds to the gp130 membrane subunit and may allow various cells to respond to IL-6 [9]. According to the ENSEMBL database (https://www.ensembl.org, November 2018) [10], the *IL6R* gene is situated on the first chromosome in the position from 154,405,193 to 154,469,450 077 in reference to assembly GRch38 on the forward strand. The SNP Asp358Ala in the *IL6R* gene (A>C; rs2228145) is associated with various diseases, such as asthma, coronary heart disease, or type 1 diabetes. In the case of RA, the C allele in this variant reduces susceptibility to the disease [11]. The SNP with rs4129267 is located at intron 8 (1q21.3) in the *IL6R* gene and has previously been reported to be associated with asthma susceptibility in Europeans [12].

The suppressor of cytokine signaling 3 (*SOCS3*) gene is located on chromosome 17 on the reverse strand in location 78,356,778–78,360. With regard to the immune response, this gene participates in negative regulation of the inflammatory process, mainly through negative regulation of the JAK-STAT cascade (ENSEMBL database (https://www.ensembl.org, November 2018) [10]. The SNP with rs4969168 in the *SOCS3* gene is located in the 3′UTR region, while *SOCS3* rs4969170 is located in the promoter. Both variants have been studied for an association with various diseases, such as atopic dermatitis [13], HCV treatment-induced neutropenia and thrombocytopenia [14], and with insulin resistance in chronic hepatitis C patients [15], but only the *SOCS3* rs4969170 polymorphism has been found to be linked to these conditions. There is insufficient information regarding possible methylation changes in both the *IL6R* and *SOSC3* genes in RA; only Liu et al. have reported a single CpG potentially associated with the RA phenotype in Supplementary Material, but this single methylation locus was not finally reported as a genetic risk factor [16].

Given that both *SOCS3* and *IL6R* play a key role in the IL-6 signaling pathway, the main aim of the current study was to investigate whether the methylation patterns of *SOCS3* and *IL6R* promoters are associated with the morbidity and severity of RA. A secondary challenge was to verify the hypothesis that SNPs in these genes may be considered genetic risk factors associated with rheumatoid factor- (RF-) positive patients.

## 2. Materials and Methods

### 2.1. Patients

A total of 146 unrelated individuals, 122 with RA and 24 healthy controls, were enrolled in the study. The Medical University in Lublin Ethics Committee approved the study (protocol number KE-0254/7/2016), and the patients provided written informed consent.

Whole blood and serum samples were collected and stored at −80°C until analysis. The study included two approaches–epigenetic and genetic. Epigenetic assessments involved analysis of *SOCS3* and *IL6* promoters methylation in subjects with high disease activity and patients in remission, as well as healthy controls. Genetic assessments involved all individuals enrolled to the study.

### 2.2. DNA Extraction and Bisulfide Conversion

DNA was extracted from 200 *μ*l of frozen whole blood according to the manufacturer's protocol using the GeneMATRIX Quick Blood DNA Purification Kit (Eurx, Poland). A 1 *μ*g DNA sample was converted by sodium bisulfite using the EZ DNA Methylation Gold Kit (Zymo Research, USA) according to the manufacturer's recommendations, but the elution volume was increased to 50 *μ*l. DNA was stored at −80°C until further analysis.

### 2.3. Methylation Study

From a total of 122 RA in the methylation study were selected 52 patients with RA as well as 24 healthy controls. Patients were chosen based on disease activity score 28 (DAS28): only individuals with high disease activity (DAS28 > 5.1; *n* = 30) and in remission (DAS28 ≤ 2.6; *n* = 22) were enrolled.

The promoter regions were found in the Eukaryotic Promoter Database (EPD, https://epd.vital-it.ch, November 2017) [17]. Using the EPD motif tool (based on JASPAR core 2018 vertebrates), in the *SOCS3* promoter region we found a predictive binding site for transcriptional repressor CTCF (the cut off *p* value was 0.0001) at position −874 with respect to the transcription start site (TSS). Primers complementary to the methylated sequence flanked this position at locations from −961 to −835 in reference to TSS. In the *IL6R* promoter, we found a CpG-rich region downstream of TSS and decided to locate the primers from −169 to −78. In this target region we found predictive binding sites for transcription factors from the family of interferon regulatory factors, IRF4 and IRF5 (the cut off *p* value was 0.001), which may play a significant role in regulation of the immune system [18]. Primers were designed using MethPrimer Software, version 1.0 [19]. Their sequences are presented in Table 1. The ability to amplify specific sequences was evaluated using fully methylated and unmethylated DNA controls (EpiTect PCR Control DNA Set, Qiagen, Germany). PCR products were visualized in a 2% agarose gel.

All samples had previously been evaluated for suitability for epigenetic testing by amplifying a DNA region free of CpG sites in the beta-actin gene (*ACTB*). Samples were considered suitable for further analysis when the cycle threshold (Ct) value was below 30. Qualitative real-time methylation-specific PCR (q-MSP) was used to analyze the methylation status. The methylation status of the promoters was evaluated using primers complementary to the methylated target sequence. The Q-MSP reaction contained 500 nM of each primer for *SOCS3*, 300 nM for both *IL6R* and *ACTB*, and 3 *μ*l or 4 *μ*l bisulfide-treated DNA for *SOCS3* or *IL6R*, respectively. The reaction was performed using ExiLENT SYBR Green master mix (EXIQON, Denmark) in the COBAS z480 Real-Time PCR System under the thermal cycling conditions given in the mix manual, in 50 amplification cycles and with an annealing/elongation step at 63°C or 62°C for *SOCS3* and *IL6R*, accordingly, for 1 min. This was followed by a PCR melt curve analysis. All reaction plates contained a positive control sample with 1% methylated sequences in 99% unmethylated sequences. The data were analyzed using the Absolute Quantification module (LightCycler 480 SW, version 1.5.1.62 SP2—UDF v.2.0.0, Roche, Germany), with the maximum second derivative selected as the calculation model. If the Ct of the sample was lower than the Ct of the positive control, the sample was assessed as positive for methylation.

### 2.4. Genotyping

Genotypes of *SOCS3* rs4969168 (G>A, assay ID: C___1706397_20, Thermo Fisher Scientific, USA) and rs4969170 (G>A, assay ID: C__29949384_10, Thermo Fisher Scientific, USA) and of *IL6R* rs4129267 (C>T, assay ID: C__26292282_10, Thermo Fisher Scientific, USA) and rs2228145 (A>C, assay ID: C__16170664_10, Thermo Fisher Scientific, USA) were ascertained by the allelic discrimination test using a TaqMan genotyping assay and the Endpoint Genotyping module of the COBAS z480 Real-Time PCR System (LightCycler 480 SW, version 1.5.1.62 SP2—UDF v.2.0.0, Roche, Germany).

### 2.5. Antibody Testing

Anticitrullinated protein antibodies (ACPA; DiaMetra, Italy) and RF (TestLine Clinical Diagnostics) were determined in serum using an enzyme-linked immunosorbent immunoassay (ELISA) and an absorbance reader (Tecan infinite M200 Pro reader and Magellan software, version 7.1). All procedures were performed according to the manufacturer's recommendations. The reference range was up to 30 U/ml for ACPA and up to 22 U/ml for RF. Antibody titers above this range were considered positive.

### 2.6. Statistical Analysis

Distribution of variables was assessed by the Shapiro–Wilk W test. Quantitative values were presented as mean ± SD or median [interquartile range]. Differences between two independent groups were compared with Student's *t*-test or the Mann–Whitney *U* test. Qualitative parameters are given as numbers with percentage and were evaluated using contingency tables with a Fisher's exact test (one-sided) or a *χ*^2^ test with Yates's correction was used. Logistic regression was used for evaluation of the odds ratio (OR) and confidence intervals (CI). A *p* value <0.05 was considered statistically significant. The analysis was performed with STATISTICA Version 13 (StatSoft Inc., USA). Linkage disequilibrium between *SOCS3* rs4969168 and rs4969170 and between *IL6R* rs4129267 and rs2228145 was evaluated by LDlink application, version 3.6 [20]; for details please refer to Supplementary Figures [Supplementary-material supplementary-material-1] and [Supplementary-material supplementary-material-1], respectively.

## 3. Results and Discussion

### 3.1. Results

Characteristics of the patients with RA and healthy controls are presented in Table 2.

#### 3.1.1. SOCS3 and IL6R Methylation

A detailed characterization of the RA groups is presented in Table 3. In the group with high disease activity, more patients were treated with steroids than in the patients in remission, which was in line in with common treatment strategies. ESR, CRP, and visual analog scores were higher, as expected, in the group with disease exacerbation than in the patients in remission.

Patients with RA and healthy controls did not differ in methylation profile for either *SOCS3* or *IL6R*. None of the samples had a Ct value below the determined cut-off value (no methylation signals were obtained in both healthy controls and patients), thus no differences in epigenetic regulation were detected. Positive control (1% of DNA methylation) had a valid signal.

#### 3.1.2. Genotyping

Detailed genotyping data, including the genetic models, are presented in Supplementary Tables [Supplementary-material supplementary-material-1]–[Supplementary-material supplementary-material-1]. The genotypes and alleles distribution of *SOCS3* and *IL6R* between patients with RA (*n* = 122) and healthy controls (*n* = 24), RF-positive (*n* = 82) and RF-negative (*n* = 40), and ACPA-positive (*n* = 104) and ACPA-negative (*n* = 18) patients are given in Supplementary Tables [Supplementary-material supplementary-material-1] and [Supplementary-material supplementary-material-1], accordingly. No differences in genotypes frequency in regard to genetic models (codominant, dominant, overdominant, and recessive) were found between patients with RA and healthy controls and between patients divided by ACPA-positivity (Supplementary Tables [Supplementary-material supplementary-material-1], [Supplementary-material supplementary-material-1], [Supplementary-material supplementary-material-1], and [Supplementary-material supplementary-material-1]). RF-positive patients had a lower frequency of genotype GA in *SOCS3* rs4969168 in overdominant genetic model (Fisher's exact test *p* value=0.0412, Supplementary [Supplementary-material supplementary-material-1]). Other models were not showing differences between RF-positive and RF-negative patients in regard to studied SNPs.

The distribution of *SOCS3* rs4969168 and rs4969170 and of *IL6R* rs4129267 and rs2228145 genotypes was in accordance with Hardy–Weinberg equilibrium (*p*=0.33, *p*=0.72, *p*=0.26, and *p*=0.62, respectively).

### 3.2. Discussion

The current study is the first to demonstrate that the morbidity and severity of RA are not associated with DNA methylation in the regulatory regions tested in either the *IL6R* or the *SOCS3* genes. Here we have shown that neither genes had more intensive methylation in the promoter region than in the healthy controls (all patients had fully unmethylated DNA or below the detection limit), which may suggest that the IL-6 signaling pathway in whole blood is regulated by other pathways. We have found only one study investigating methylation patterns in RA involving both the *IL6R* and *SOCS3* genes [16]. Liu et al. reported only one CpG site in *SOCS3* and four CpG sites in *IL6R* associated with an RA phenotype; however, these loci were not ultimately reported as genetic risk factors for RA [16]. *SOCS3* negatively regulates inflammation via the transcriptional factor (TF) STAT3, especially under the influence of cytokines using gp130 receptors, such as IL-6, IL-11, and IL-27 [21]. Liu et al. [16] reported only a single CpG site about 83 bp upstream of the beginning of our target region. We designed the primers in positions from −961 to −835 in reference to TSS, and they flanked a region that may be a binding site for TF CTCF, a multifunctional molecule involved in transcription, imprinting, and long-distance chromatin interaction. The negative correlation between CTCF binding and DNA methylation has been well demonstrated [22], and so the choice of this region in the promoter was not random. Moreover, a mechanism in which IL-6 increases the activity of DNA methyltransferase-1 and leads to *SOCS3* promoter hypermethylation has previously been reported in ulcerative colitis patients [23]. We believe that *SOCS3* may be epigenetically regulated by DNA methylation, but it is important to find the specific region responsible for gene expression. Yoshikawa et al. [24] have described a region between −556 and −335 bp relative to TSS where hypermethylation was detected and demonstrated that this location may be a binding site for microRNA-122, leading to decreased expression of *SOCS3*. Binding sites for TF STAT3 are also situated at the micro-RNA-122 binding site, and thus binding micro-RNA may block activity of STAT3, which may prove critical for regulation of this gene. On the other hand, Yoshikawa et al. have shown only one possible mechanism of *SOCS3* regulation. They worked with material from the liver and showed regulation induced by microRNA-122, which is specific for this tissue. It is possible that the use of more specific tissue material such as the synovium might provide more interesting data. Additional research on DNA methylation is unquestionably needed, but in our opinion epigenetic studies should be more targeted at interaction between DNA methylation and microRNAs, because this combination may provide more valuable data. Moreover, Isomaki et al. [25] have reported that *SOCS* family expression is profoundly changed in patients with RA but found that the level of gene expression was cell population and cellular compartment. With regard to *SOCS3*, they found increased expression of *SOCS3* mainly in peripheral blood monocytes from subjects with RA relative to healthy controls. Isomäki et al. used peripheral blood mononuclear cells as working material, while we have used frozen whole blood, and although we achieved a limit of detection of about 1%, we found no differences between patients and controls. Moreover, we also found no differences in the frequency of either SNP in the *SOCS3* gene. To the best of our knowledge, these SNPs have never been tested with regard to RA in Caucasians. We found a lower frequency of genotype GA in *SOCS3* rs4969168 in a overdominant model in RF-positive patients. Fang et al. [26] reported that genotype AG in rs4969168 showed the significant association with infantile asthma. Mentioned variant has had a higher frequency in patients compared to controls and genotypes AG and GG were associated with increased risk of asthma development. Both RA and asthma have a different pathogenesis; however it may be important to pay attention to genetic models analysis and their association with disease. Yan et al. [27] reported potential biological function of *SOCS3* rs4969170. Discussed SNP was associated with elevated mRNA and protein expression in the patients with Graves' disease. They found that individuals with AA genotype have had an increased levels of mRNA and protein compared to carriers of GG genotype. Similar finding are demonstrated by Zheng et al. [15]. They reported increased levels of *SOCS3* mRNA in insulin-resistant carriers of genotype AA. Both SNPs, rs4969168 and 4969179, are in linkage disequilibrium. Due to limited number of individuals haplotype analysis was out of the scope of this study; however this fact may be useful for further studies. Taken together, we found that SNPs with rs4969168 and rs4969170 may not be useful molecular markers related to RA.

DNA methylation in the *SOCS3* gene may also not be useful as an epigenetic marker of disease in whole blood, but perhaps a focus on other epigenetic regulations, such as microRNA expression or histone modification in relation to this gene, may provide more valuable information.

The *IL6R* gene has long been a subject of interest among researchers, especially in the context of potential therapy using IL-6R antibodies [28]. For the *IL6R* gene we designed primers flanking the important regulatory region for TF IRF5. IRF5 is an important molecule responsible for transcription [18] and is associated with acute and chronic inflammation [29] as well as probability of being considered as epigenetic marker of RA [30]. In this gene we also found no differences between patients with RA and controls; all patients had fully unmethylated DNA. In contrast, de la Rica et al. [28] have found differences in the methylation pattern between DNA from rheumatoid arthritis synovial fibroblasts (RASFs) and osteoarthritis synovial fibroblasts (OASFs). Those from RA were hypomethylated in RASFs with respect to OASFs. IL-6R and its ligand play a key role in RA pathogenesis, including joint destruction, so it may be more useful to investigate the differences in DNA methylation in synovial tissue compared to in whole blood, as was done in our study. Moreover, the lack of differences in whole blood may be explained by the fact that IL-6R is distributed on the surface of most cells, and regulation of its expression in whole blood may take place via other pathways. Limited proteolysis of IL-6R or alternative mRNA splicing may generate a sIL-6R, which may induce an IL-6 transsignaling process [8, 9]. Furthermore, Liu et al. [16] have found four CpG sites associated with the RA phenotype, which may suggest a possible impact of *IL6R* gene methylation in RA. That study focused on finding differences between single CpG sites. In our study we have focused on finding a critical regulatory region, such as a TF binding site. However, both our study and that conducted by Liu et al. were performed on whole blood, which we considered essential for later clinical use, as not every laboratory has the capacity to test material such as synovial tissue. Further DNA methylation testing between specific cells and tissue, as well as in different methylation loci, is necessary to better understand the role of epigenetic regulations in *IL6R* expression. We also found no differences in the frequency of either SNP between RF-positive and RF-negative patients and ACPA-positive and ACPA-negative patients. Previous reports [11, 31] have shown a possible association between IL6R polymorphisms and susceptibility to RA, as well as joint destruction in subjects with RA. Biological function of rs2228145 was reported by Garbers et al. [32]. Variant allele was associated with twofold increased level of sIL6R in serum and was related to reduced production of CRP induced by IL6. Moreover, van Dongen et al. [11] reported that C allele reduces susceptibility to RA. Revez et al. [33] confirmed the impact of rs2228145 on increased levels of sIL6R. Moreover, they reported that each copy of T allele in rs4129267 increased the level of serum sIL6. Both SNPs were also in a strong linkage disequilibrium. As was mentioned above, haplotype analysis was out of scope of this study, but additional analysis related to these SNPs is required. To the best of our knowledge, we have demonstrated for the first time a lack of association between these SNPs with rs4129267 and rs2228145 and RF-positivity and ACPA-positivity. It is difficult to compare our data with previous reports, and the power of analysis of our study is limited by the small group size.

A major limitation of determination of DNA methylation is the test material. We tested frozen whole blood and we used a single primer set to evaluate potential relationships between RA development and DNA methylation. A deeper analysis could be performed by pyrosequencing of a whole *SOCS3* and *IL6R* promoters or analysis of sorted blood mononuclear cells subpopulations. Also methylation analysis of more specific tissue, like synovial may provide additional information about epigenetic regulation of *SOCS3* and *IL6R* genes. It is important to mention that our study shows a basic data with low number of individuals enrolled to genotyping; thus interpretation of the results is limited (please refer to confidence intervals in Supplementary Tables [Supplementary-material supplementary-material-1]–[Supplementary-material supplementary-material-1] and [Supplementary-material supplementary-material-1]–[Supplementary-material supplementary-material-1]). Genotyping analysis on a larger cohort is necessary to confirm our data. Despite that data shown in the manuscript were an initial data, they may stimulate further studies.

## 4. Conclusions

To conclude, *SOCS3* and *IL6R* are critical genes involved in the IL-6 signaling pathway. In whole blood, no methylation signals were obtained from both genes. Deeper analysis of more specific material, including subpopulations of blood mononuclear cells or synovial tissue, may be useful to determine their methylation status and relationships with RA.

## Figures and Tables

**Table 1:** Characteristics of primers and amplicons.

Gene	Primer name	Sequence 5′ ⟶ 3′^*∗*^	Amplicon size [bp]	Amplicon location with reference to assembly GRCh38 [chromosome : start : end : strand]	Primer complementary to methylated/unmethylated sequences
*ACT-B*	ACTB sense	GGTGGTGATGGAGGAGGTTTAG	115	7 : 5532117 : 5532232 : −1	Independent of methylation status
	ACTB antisense	CCCTTAAAAATTACAAAAACCACAACC			

*SOCS3*	SOCS3_ methyl_sense	GTGGAA**CG**ATGGTTTTAATTTA**CG**	126	17 : 78360912 : 78361038 : 1	Methylated sequences
	SOCS3_ methyl_antisense	ATTCC**CG**CAAATCCCTAA**CG**			
	SOCS3_ unmethyl_sense	GGTGGAATGATGGTTTTAATTTATG	127	17 : 78360911 : 78361038 : 1	Unmethylated sequences
	SOCS3_ unmethyl_antisense	ATTCCCACAAATCCCTAACATAC			

*IL6R*	IL6R_ methyl_sense	**CG**TATTTTGGGA**CG**GTTTAGAGA**C**	91	1 : 154405299 : 154405390 : 1	Methylated sequences
	IL6R_ methyl_antisense	CACATAACTCAAAA**CG**A**CG**AA**CG**			
	IL6R_ unmethyl_sense	TTGTATTTTGGGATGGTTTAGAGATG	95	1 : 154405298 : 154405393 : 1	Unmethylated sequences
	IL6R_ unmethyl_antisense	TCACACATAACTCAAAACAACAAACAAT			

^*∗*^CpG sites in primer sequence are in bold.

**Table 2:** Characteristics of patients with rheumatoid arthritis and healthy controls enrolled to the study.

Characteristics	RA overall, *n* = 122	HC, *n* = 24	*p* value
Age (years)	52.2 ± 12.3	52.9 ± 8.6	0.99
Females; *n* (%)	103 (84.4)	18 (75)	0.41
Disease duration (years)	10 [3–16]	n/a	n/a
RF-positive; *n* (%),	82 (67.2)	None	n/a
ACPA-positive; *n* (%)	104 (85.2)	None	n/a
ESR	26 [8–57.5]	15 [7–19]	**0.001**
CRP (mg/dl)	8.54 [0.53–19.12]	0.58 [0.19–1.97]	**<0.0001**
DAS28	3.95 ± 1.55	n/a	n/a
Swollen joints	1 [0–4]	n/a	n/a
Tender joints	3 [1–6]	n/a	n/a
VAS PGA	27 [9–61]	n/a	n/a
VAS PhGA	20 [7–49]	n/a	n/a

Data are presented as mean ± SD, number (percentage), or median [interquartile range]. ACPA, anticitrullinated protein antibodies; DAS28, disease activity score 28; CRP, C-reactive protein; cDMARD, classical disease-modifying antirheumatic drugs; ESR, erythrocyte sedimentation rate; RF, rheumatoid factor; VAS PhGA, visual analog scale physician global assessments; VAS PGA, visual analog scale patient global assessments.

**Table 3:** Characteristics of patients with rheumatoid arthritis included in the methylation analysis.

Characteristics	High disease activity, *n* = 30	Remission, *n* = 22	*p* value
Age (years)	53.6 ± 12.3	48.7 ± 12.7	0.21
Females; *n* (%)	22 (73.3)	21 (95.5)	0.09
Disease duration (years)	10 [2–16]	9.5 [3.5–16]	0.96
RF-positive; *n* (%),	23 (76.7)	12 (54.5)	0.17
ACPA-positive; *n* (%)	29 (96.7)	20 (90.9)	0.78
ESR	54 [28–67]	7 [2–11.5]	**<0.0001**
CRP (mg/dl)	15.72 [8.54–27.2]	0.45 [0.13–4.09]	**<0.0001**
VAS PGA	67.5 [52–73]	8 [3–9.5]	**<0.0001**
VAS PhGA	59 [49–70]	5 [1.5–7]	**<0.0001**
Treatment:			
At least on methotrexate, *n* (%)	23 (76.7)	15 (68.2)	0.72
At least on biologics, *n* (%)	7 (23.3)	11 (50)	0.09
At least on steroids, *n* (%)	23 (76.7)	7 (31.8)	**0.003**
Single-line therapy, *n* (%)	**4 (13.3)**	**7 (31.8)**	0.2
Methotrexate, *n* (%)	2 (6.7)	3 (13.6)	0.71
Biologics, *n* (%)	0	1 (4.5)	0.88
Steroids, *n* (%)	1 (3.3)	0	0.88
Other cDMARD, *n* (%)	1 (3.3)	3 (13.6)	0.39
Double-line therapy, *n* (%)	**17 (56.7)**	**10 (45.5)**	0.6
Methotrexate + steroids, *n* (%)	13 (43.3)	1 (4.5)	**0.0051**
Methotrexate + biologics, *n* (%)	2 (6.7)	4 (18.2)	0.4
Steroids + biologics, *n* (%)	0	1 (4.5)	0.88
Other, *n* (%)	2 (6.7)	4 (18.2)	0.4
Triple-line therapy, *n* (%)	**9 (30)**	**5 (22.7)**	0.79

Data are presented as mean ± SD, number (percentage), or median [interquartile range]. ACPA, anticitrullinated protein antibodies; CRP, C-reactive protein; cDMARD, classical disease-modifying antirheumatic drugs; ESR, erythrocyte sedimentation rate; RF, rheumatoid factor; VAS PhGA, visual analog scale physician global assessments; VAS PGA, visual analog scale patient global assessments.

## Data Availability

The data used to support the findings of this study are available from the corresponding author upon request.

## Supplementary Materials

Supplementary Materials Table S1: genotypes and alleles distribution. Table S2: distribution of genotypes in different genetic models between patients and controls. Table S3: distribution of genotypes in different genetic models between rheumatoid factor positive and rheumatoid factor negative patients. Table S4: distribution of genotypes in different genetic models between anticitrullinated protein antibodies positive and anticitrullinated protein antibodies negative patients. Table S5: genotypes and alleles distribution. Table S6: distribution of genotypes in different genetic models between patients and controls. Table S7: distribution of genotypes in different genetic models between rheumatoid factor positive and rheumatoid factor negative patients. Table S8: distribution of genotypes in different genetic models between anticitrullinated protein antibodies positive and anticitrullinated protein antibodies negative patients. Figure S1: linkage disequilibrium between rs4969168 and rs4969170. Figure S2: linkage disequilibrium between rs4129267 and rs2228145.
