# Supplementary material for: Methylation Pattern of the SOCS3 and IL6R Promoters in Rheumatoid Arthritis
Source: Int J Inflam. 2020 Mar 31;2020:8394659. doi: 10.1155/2020/8394659 (PMC7150677; doi:10.1155/2020/8394659)
Supplement: Supplementary Materials — Table S1: genotypes and alleles distribution. Table S2: distribution of genotypes in different genetic models between patients and controls. Table S3: distribution of genotypes in different genetic models between rheumatoid factor positive and rheumatoid factor negative patients. Table S4: distribution of genotypes in different genetic models between anticitrullinated protein antibodies positive and anticitrullinated protein antibodies negative patients. Table S5: genotypes and alleles distribution. Table S6: distribution of genotypes in different genetic models between patients and controls. Table S7: distribution of genotypes in different genetic models between rheumatoid factor positive and rheumatoid factor negative patients. Table S8: distribution of genotypes in different genetic models between anticitrullinated protein antibodies positive and anticitrullinated protein antibodies negative patients. Figure S1: linkage disequilibrium between rs4969168 and rs4969170. Figure S2: linkage disequilibrium between rs4129267 and rs2228145. [file 8394659.f1.pdf]

Supplementary data

Methylation pattern of the *SOCS3* and *IL6R* promoters in rheumatoid arthritis.

Cieśla M. et al.

Table S1. Genotypes and alleles distribution.

***SOCS3* SNPs**		Number of carriers (%)						
**rs4969168**	**Allele/ Genotype**	**RA RF-positive, n = 82**	**RA RF-negative, n = 40**	**RA ACPA-positive, n=104**	**RA ACPA-negative, n=18**	**RA overall, n = 122**	**Controls, n = 24**	**Subjects in a methylation study, n = 76**
	**MAF (A allele)**	11%	15%	12.5%	11.1%	12.3%	10.4%	6.6%
	GG	67 (81.7)	28 (70)	81 (77.9)	14 (77.8)	95 (77.9)	19 (79.2)	66 (86.8)
	GA	12 (14.6)	12 (30)	20 (19.2)	4 (22.2)	24 (19.7)	5 (20.8)	10 (13.2)
	AA	3 (3.7)	0	3 (2.9)	0	3 (2.5)	0	0
**rs4969170**	**Allele/Genotype**							
	**MAF (A allele)**	29.9%	35%	30.3%	38.9%	31.6%	45.8%	30.9%
	GG	41 (50)	17 (42.5)	51 (49)	7 (38.9)	58 (47.5)	7 (29.2)	36 (47.4)
	GA	33 (40.2)	18 (45)	43 (41.3)	8 (44.4)	51 (41.8)	12 (50)	33 (43.4)
	AA	8 (9.8)	5 (12.5)	10 (9.6)	3 (16.7)	13 (10.7)	5 (20.8)	7 (9.2)

Abbreviations: ACPA; Anti-citrullinated protein antibodies; MAF, minor allele frequency; RA, rheumatoid arthritis patients; RF, rheumatoid factor; SNP, single nucleotide polymorphism; *SOCS3*; suppressor of cytokine signaling 3 gene.

Table S2. Distribution of genotypes in a different genetic models between patients and controls.

SOCS3 **SNPs**	**Model**	**RA, n=122**	**Controls, n=24**	**OR [95% CI]**	**p-value**	**Fisher's exact p-value**
**rs4969168**	**Codominant 1** GG vs. GA	95 (77.9) vs. 24 (19.7)	19 (79.2) vs. 5 (20.8)	0.96 [0.32-2.87]	0.94	0.57
	**Codominant 2** GG vs. AA	95 (77.9) vs. 3 (2.5)	19 (79.2) vs. 0	nd.		0.58
	**Dominant** GA+AA vs. GG	27 (22.1) vs. 95 (77.9)	5 (20.8) vs. 19 (79.2)	0.93 [0.31-2.74]	0.89	0.56
	**Overdominant** GA vs. GG+AA	24 (19.7) vs. 98 (80.3)	5 (20.8) vs. 19 (79.2)	0.93 [0.31-2.76]	0.9	0.54
	**Recessive** AA vs. GA+GG	3 (2.5) vs. 119 (97.5)	0 vs. 24 (100)	nd.		0.58
**rs4969170**						
	**Codominant 1** GG vs. GA	58 (47.5) vs. 51 (41.8)	7 (29.2) vs. 12 (50)	0.51 [0.19-1.42	0.19	0.14
	**Codominant 2** GG vs. AA	58 (47.5) vs. 13 (10.7)	7 (29.2) vs. 5 (20.8)	0.31 [0.08-1.17]	0.09	0.08
	**Dominant** GA+AA vs. GG	64 (52.5) vs.58 (47.5)	17 (70.8) vs. 7 (29.2)	0.45 [0.17-1.18]	0.09	0.07
	**Overdominant** GA vs. GG+AA	51 (41.8) vs. 71 (58.2)	12 (50) vs. 12 (50)	0.72 [0.3-1.74]	0.46	0.3
	**Recessive** AA vs. GA+GG	13 (10.7) vs. 109 (89.3)	5 (20.8) vs. 19 (79.2)	0.45 [0.14-1.43]	0.19	0.15

Data are presented as number (%). Abbreviations: CI, confidence interval; OR, odds ratio. For other please refer to Table S1 in supplementary file.

Table S3. Distribution of genotypes in a different genetic models between rheumatoid factor positive and rheumatoid factor negative patients.

***SOCS3* SNPs**	**Model**	**RF positive, n=82**	**RF negative, n=40**	**OR [95% CI]**	**p-value**	**Fisher's exact p-value**
**rs4969168**	**Codominant 1** GG vs. GA	67 (81.7) vs. 12 (14.6)	28 (70) vs. 12 (30)	2.39 [0.95-6.02]	0.06	0.0504
	**Codominant 2** GG vs. AA	67 (81.7) vs. 3 (3.7)	28 (70) vs. 0	nd.		0.36
	**Dominant** GA+AA vs. GG	15 (18.3) vs. 67 (81.7)	12 (30) vs. 28 (70)	0.52 [0.22-1.27]	0.15	0.11
	**Overdominant** GA vs. GG+AA	12 (14.6) vs. 70 (85.4)	12 (30) vs. 28 (70)	0.4 [0.16-1.00]	0.05014	**0.0412**
	**Recessive** AA vs. GA+GG	3 (3.7) vs. 79 (96.3)	0 vs. 40 (100)	nd.		0.30
**rs4969170**						
	**Codominant 1** GG vs. GA	41 (50) vs. 33 (40.2)	17 (42.5) vs. 18 (45)	1.32 [0.58-2.97]	0.50	0.32
	**Codominant 2** GG vs. AA	41 (50) vs. 8 (9.8)	17 (42.5) vs. 5 (12.5)	1.51 [0.42-5.39]	0.53	0.37
	**Dominant** GA+AA vs. GG	41 (50) vs. 41 (50)	23 (57.5) vs. 17 (42.5)	0.74 [0.34-1.6]	0.44	0.28
	**Overdominant** GA vs. GG+AA	33 (40.2) vs. 49 (59.8)	18 (45) vs. 22 (55)	0.82 [0.38-1.78]	0.62	0.38
	**Recessive** AA vs. GA+GG	8 (9.8) vs. 74 (90.2)	5 (12.5) vs. 35 (87.5)	0.76 [0.23-2.51]	0.65	0.43

Data are presented as number (%). Abbreviations: please refer to Tables S1 and S2 in supplementary file.

Table S4. Distribution of genotypes in a different genetic models between anti-citrullinated protein antibodies positive and anti-citrullinated protein antibodies negative patients.

***SOCS3* SNPs**	**Model**	**ACPA positive, n=104**	**ACPA negative, n=18**	**OR [95% CI]**	**p-value**	**Fisher's exact p-value**
**rs4969168**	**Codominant 1** GG vs. GA	81 (77.9) vs. 20 (19.2)	14 (77.8) vs. 4 (22.2)	0.86 [0.25-2.95]	0.82	0.51
	**Codominant 2** GG vs. AA	81 (77.9) vs. 3 (2.9)	14 (77.8) vs. 0	nd.		0.63
	**Dominant** GA+AA vs. GG	23 (22.1) vs. 81 (77.9)	4 (22.2) vs. 14 (77.8)	0.99 [0.32-3.09]	0.99	0.6
	**Overdominant** GA vs. GG+AA	20 (19.2) vs. 84 (80.8)	4 (22.2) vs. 14 (77.8)	0.83 [0.24-2.84]	0.77	0.49
	**Recessive** AA vs. GA+GG	3 (2.9) vs. 101 (97.1)	0 vs. 18 (100)	nd.		0.62
**rs4969170**						
	**Codominant 1** GG vs. GA	51 (49) vs. 43 (41.3)	7 (38.9) vs. 8 (44.4)	0.74 [0.24-2.23]	0.58	0.39
	**Codominant 2** GG vs. AA	51 (49) vs. 10 (9.6)	7 (38.9) vs. 3 (16.7)	0.46 [0.1-2.13]	0.33	0.26
	**Dominant** GA+AA vs. GG	53 (51) vs. 51 (49)	11 (61.1) vs. 7 (38.9)	0.66 [0.24-1.86]	0.42	0.3
	**Overdominant** GA vs. GG+AA	43 (41.3) vs. 61 (58.7)	8 (44.4) vs. 10 (55.6)	0.88 [0.32-2.44]	0.81	0.5
	**Recessive** AA vs. GA+GG	10 (9.6) vs. 94 (90.4)	3 (16.7) vs. 15 (83.3)	0.53 [0.13-2.19]	0.4	0.29

Data are presented as number (%). Abbreviations: please refer to Tables S1 and S2 in supplementary file.

Table S5. Genotypes and alleles distribution.

***IL6R* SNPs**		Number of carriers (%)						
**rs4129267**	**Allele/ Genotype**	**RA RF-positive, n = 82**	**RA RF-negative, n = 40**	**RA ACPA-positive, n=104**	**RA ACPA-negative, n=18**	**RA overall, n = 122**	**Controls, n = 24**	**Subjects in a methylation study, n = 76**
	**MAF (T allele)**	29.9%	25%	29.8%	19.4%	28.3%	33.3%	30.9%
	CC	43 (52.4)	22 (55)	54 (51.9)	11 (61.1)	65 (53.3)	11 (45.8)	37 (48.7)
	CT	29 (35.4)	16 (40)	38 (36.5)	7 (38.9)	45 (36.9)	10 (41.7)	31 (40.8)
	TT	10 (12.2)	2 (5)	12 (11.5)	0	12 (9.8)	3 (12.5)	8 (10.5)
**rs2228145**	**Allele/ Genotype**							
	**MAF (C allele)**	30.5%	26.3%	30.3%	22.2%	29.1%	31.3%	32.2%
	AA	43 (52.4)	22 (55)	54 (51.9)	11 (61.1)	65 (53.3)	12 (50)	37 (48.7)
	AC	28 (34.1)	15 (37.5)	37 (35.6)	6 (33.3)	43 (35.2)	9 (37.5)	29 (38.2)
	CC	11 (13.4)	3 (7.5)	13 (12.5)	1 (5.6)	14 (11.5)	3 (12.5)	10 (13.2)

Abbreviations: *IL6R*; Interleukin-6 receptor gene. For other please refer to Table S1 in supplementary file.

Table S6. Distribution of genotypes in a different genetic models between patients and controls.

IL6R SNPs	Model	RA overall, n=122	Controls, n=24	OR (95% CI)	p-value	Fisher's exact p-value
**rs4129267**	**Codominant 1** CC vs. CT	65 (53.3) vs. 45 (36.9)	11 (45.8) vs. 10 (41.7)	0.76 [0.3-1.96]	0.57	0.37
	**Codominant 2** CC vs. TT	65 (53.3) vs. 12 (9.8)	11 (45.8) vs. 3 (12.5)	0.68 [0.16-2.84]	0.6	0.42
	**Dominant** CT+TT vs. CC	57 (46.7) vs. 65 (53.3)	13 (54.2) vs. 11 (45.8)	0.74 [0.31-1.8]	0.5	0.33
	**Overdominant** CT vs. CC+TT	45 (36.9) vs. 77 (63.1)	10 (41.7) vs. 14 (58.3)	0.82 [0.33-2.01	0.66	0.41
	**Recessive** TT vs. CC+CT	12 (9.8) vs. 110 (90.2)	3 (12.5) vs. 21 (87.5)	0.76 [0.2-2.98]	0.7	0.46
**rs2228145**						
	**Codominant 1** AA vs. AC	65 (53.3) vs. 43 (35.2)	12 (50) vs. 9 (37.5)	0.88 [0.34-2.29]	0.8	0.49
	**Codominant 2** AA vs. CC	65 (53.3) vs. 14 (11.5)	12 (50) vs. 3 (12.5)	0.86 [0.21-3.53]	0.84	0.54
	**Dominant** AC+CC vs. AA	57 (46.7) vs. 65 (53.3)	12 (50) vs. 12 (50)	0.88 [0.36-2.12]	0.77	0.47
	**Overdominant** AC vs. AA+CC	43 (35.2) vs. 79 (64.8)	9 (37.5) vs. 15 (62.5)	1.1 [0.44-2.75]	0.83	0.5
	**Recessive** CC vs. AA+AC	14 (11.5) vs. 108 (88.5)	3 (12.5) vs. 21 (87.5)	0.91 [0.24-3.48]	0.89	0.56

Data are presented as number (%). Abbreviations: please refer to Tables S1, S2 and S5 in supplementary file.

Table S7. Distribution of genotypes in a different genetic models between rheumatoid factor positive and rheumatoid factor negative patients.

IL6R SNPs	Model	RF positive, n=82	RF negative, n=40	OR [95% CI]	p-value	Fisher's exact p-value
**rs4129267**	**Codominant 1** CC vs. CT	43 (52.4) vs. 29 (35.4)	22 (55) vs. 16 (40)	1.08 [0.48-2.42]	0.85	0.51
	**Codominant 2** CC vs. TT	43 (52.4) vs. 10 (12.2)	22 (55) vs. 2 (5)	0.39 [0.08-1.99]	0.22	0.20
	**Dominant** CT+TT vs. CC	39 (47.6) vs. 43 (52.4)	18 (45) vs. 22 (55)	1.11 [0.51-2.39]	0.79	0.47
	**Overdominant** CT vs. CC+TT	29 (35.4) vs. 53 (64.6)	16 (40) vs. 24 (60)	0.82 [0.37-1.8]	0.62	0.38
	**Recessive** TT vs. CC+CT	10 (12.2) vs. 72 (87.8)	2 (5) vs. 38 (95)	2.64 [0.54-12.87]	0.19	0.18
**rs2228145**						
	**Codominant 1** AA vs. AC	43 (52.4) vs. 28 (34.1)	22 (55) vs. 15 (37.5)	1.05 [0.46-2.38]	0.91	0.54
	**Codominant 2** AA vs. CC	43 (52.4) vs. 11 (13.4)	22 (55) vs. 3 (7.5)	0.53 [0.13-2.16]	0.35	0.28
	**Dominant** AC+CC vs. AA	39 (47.6) vs. 43 (52.4)	18 (45) vs. 22 (55)	1.11 [0.51-2.39]	0.79	0.47
	**Overdominant** AC vs. AA+CC	28 (34.1) vs. 54 (65.9)	15 (37.5) vs. 25 (62.5)	0.86 [0.39-1.91]	0.72	0.43
	**Recessive** CC vs. AA+AC	11 (13.4) vs. 71 (86.6)	3 (7.5) vs. 37 (92.5)	1.91 [0.5-7.38]	0.32	0.26

Data are presented as number (%). Abbreviations: please refer to Tables S1, S2 and S5 in supplementary file.

Table S8. Distribution of genotypes in a different genetic models between anti-citrullinated protein antibodies positive and anti-citrullinated protein antibodies negative patients.

***IL6R* SNPs**	**Model**	**ACPA positive, n=104**	**ACPA negative, n=18**	**OR [95% CI]**	**p-value**	**Fisher's exact p-value**
**rs4129267**	**Codominant 1** CC vs. CT	54 (51.9) vs. 38 (36.5)	11 (61.1) vs. 7 (38.9)	1.11 [0.39-3.15]	0.85	0.53
	**Codominant 2** CC vs. TT	54 (51.9) vs. 12 (11.5)	11 (61.1) vs. 0	nd.		0.13
	**Dominant** CT+TT vs. CC	50 (48.1) vs. 54 (51.9)	7 (38.9) vs. 11 (61.1)	1.46 [0.52-4.09]	0.47	0.32
	**Overdominant** CT vs. CC+TT	38 (36.5) vs. 66 (63.5)	7 (38.9) vs. 11 (61.1)	0.9 [0.32-2.56]	0.85	0.52
	**Recessive** TT vs. CC+CT	12 (11.5) vs. 92 (88.5)	0 vs. 18 (100)	nd.		0.13
**rs2228145**						
	**Codominant 1** AA vs. AC	54 (51.9) vs. 37 (35.6)	11 (61.1) vs. 6 (33.3)	0.8 [0.27-2.37]	0.68	0.45
	**Codominant 2** AA vs. CC	54 (51.9) vs. 13 (12.5)	11 (61.1) vs. 1 (5.6)	0.38 [0.04-3.3]	0.32	0.32
	**Dominant** AC+CC vs. AA	50 (48.1) vs. 54 (51.9)	7 (38.9) vs. 11 (61.1)	1.46 [0.52-4.09]	0.47	0.32
	**Overdominant** AC vs. AA+CC	37 (35.6) vs. 67 (64.4)	6 (33.3) vs. 12 (66.7)	1.1 [0.38-3.22]	0.85	0.54
	**Recessive** CC vs. AA+AC	13 (12.5) vs. 91 (87.5)	1 (5.6) vs. 17 (94.4)	2.43 [0.29-20.24	0.36	0.35

Data are presented as number (%). Abbreviations: please refer to Tables S1, S2 and S5 in supplementary file.

Figure S1. Linkage disequilibrium between rs4969168 and rs4969170.

Query SNPs:

rs4969168 (chr17:76353793)

rs4969170 (chr17:76360538)

CEU Haplotypes:

```
              rs4969170
              A       G
            -----------------
          A | 25    | 1     | 26    (0.131)
rs4969168   -----------------
          G | 43    | 129   | 172   (0.869)
            -----------------
              68      130     198
             (0.343) (0.657)
```

G_G: 129 (0.652)

G_A: 43 (0.217)

A_A: 25 (0.126)

A_G: 1 (0.005)

D': 0.9414

R2: 0.2561

Chi-sq: 50.7121

p-value: <0.0001

rs4969168(A) allele is correlated with rs4969170(A) allele

rs4969168(G) allele is correlated with rs4969170(G) allele

Figure S2. Linkage disequilibrium between rs4129267 and rs2228145.

```
Query SNPs:
rs4129267 (chr1:154426264)
rs2228145 (chr1:154426970)

CEU Haplotypes:
             rs2228145
             A       C
           -----------------
         C | 129   | 0     | 129   (0.652)
rs4129267  -----------------
         T | 0     | 69    | 69    (0.348)
           -----------------
             129     69      198
            (0.652) (0.348)
        C_A: 129 (0.652)
        T_C: 69 (0.348)
        C_C: 0 (0.0)
        T_A: 0 (0.0)
        D': 1.0
        R2: 1.0
     Chi-sq: 198.0
    p-value: <0.0001
rs4129267(C) allele is correlated with rs2228145(A) allele
rs4129267(T) allele is correlated with rs2228145(C) allele
```
